# Mycotoxins as an Underestimated Honeybee Stressor: Aflatoxin, Contaminated Pollen, and Colony-Level Risk

**DOI:** 10.3390/biology15131027

**Published:** 2026-06-27

**Authors:** Zunair Ahsan, Mokhtar Rejili, Kang Wang

**Affiliations:** 1 Institute of Apiculture Research, College of Animal Science and Technology, Yangzhou University, 88 South University Rd., Yangzhou 225009, China; mh24057@stu.yzu.edu.cn; 2 Department of Biology, College of Sciences, Imam Mohammad Ibn Saud Islamic University (IMSIU), Riyadh 11623, Saudi Arabia; msrejili@imamu.edu.sa

**Keywords:** aflatoxin B1, *Apis mellifera*, bee bread, colony stress, fungal toxins, honeybee, mycotoxins, nutritional toxicology, pollen contamination, pollinator health

## Abstract

Pollinators play a critical role in agricultural productivity and the maintenance of flowering plant diversity, yet their health is increasingly threatened by multiple environmental stressors. While research has traditionally focused on pathogens, pesticides, habitat loss, and nutritional limitation, fungal secondary metabolites, mycotoxins, remain an underappreciated risk factor. This review synthesizes current knowledge on the presence, exposure pathways, and biological impacts of key mycotoxins, including aflatoxin B1, ochratoxin A, deoxynivalenol, zearalenone, and T-2 toxin, in bee-collected pollen and bee bread. We discuss how contaminated food matrices act as reservoirs of chronic exposure, linking forager activity, nurse bee physiology, brood development, and colony-level outcomes. Evidence from laboratory studies highlights sublethal effects on survival, hypopharyngeal gland development, immunity, and gut microbiota, with potential interactions with pathogens, nutritional stress, pesticides, and climate change. Furthermore, we extend these insights to wild pollinators, emphasizing differences in colony size, diet breadth, and detoxification capacity. Analytical methods for detecting mycotoxins, including HPLC, LC-MS/MS, and ELISA, are evaluated in terms of sensitivity, specificity, and relevance to field exposure. By integrating environmental concentrations with laboratory toxicity thresholds, this review identifies critical knowledge gaps and proposes a mechanistic framework linking mycotoxin exposure to colony-level risk. The findings underscore the need for targeted monitoring, improved risk assessment, and multi-stressor evaluation to safeguard both managed and wild pollinator populations.

## Simple Summary

A variety of factors, including diseases, pesticide exposure, and limited floral resources, contribute to declines in pollinator populations such as honeybees. Mycotoxins, secondary metabolites produced by fungi, represent an underappreciated stressor that can contaminate pollen. Foragers may collect pollen containing fungal toxins, particularly aflatoxins from *Aspergillus* species, which is subsequently stored in the hive as bee bread. This stored food is consumed primarily by nurse bees providing for developing larvae. Evidence indicates that aflatoxin exposure can impair hypopharyngeal gland development, reduce adult bee survival, and alter immune responses and gut microbiota. While mycotoxins alone are unlikely to cause colony collapse, they may exacerbate vulnerability when combined with other stressors, including poor nutrition, pesticides, and pathogens. This review examines the mechanisms of mycotoxin entry into colonies and their physiological and colony-level impacts, and emphasizes the need to integrate mycotoxins into pollinator health research, particularly under conditions where climate change may increase fungal contamination risks.

## 1. Introduction

Bees are important pollinating insects in both agricultural and natural ecosystems, where they contribute to crop yield, crop quality, and the reproduction of flowering plants [1,2]. The value of *Apis mellifera* in pollination is closely linked to its colony-level biology. A honeybee colony functions as a coordinated foraging unit rather than as isolated insects. Thousands of workers can leave the nest, locate floral patches, collect nectar and pollen, and return repeatedly to profitable flowers. This social organization allows honeybees to exploit short flowering periods more effectively than many solitary insects. Their waggle dance also supports recruitment by transferring spatial information about food resources to nestmates, which helps colonies distribute foragers across changing floral landscapes [3]. Honeybees also show behavioral traits that improve pollen movement between flowers of the same plant species. Individual foragers often remain constant to one floral type during a foraging trip, especially when floral rewards are high and easy to recognize. This behavior can reduce pollen loss between unrelated plant species and increase the chance that compatible pollen reaches receptive stigmas [4]. At the same time, honeybee colonies remain flexible at the group level. Different workers can specialize in nectar or pollen collection, and this division of labor helps colonies respond to the nutritional and energetic demands of brood rearing and adult maintenance [5,6]. Recent field studies show that colony strength and hive placement can influence honeybee visitation patterns, although higher visitation does not always translate directly into improved pollination outcomes in every crop system [7]. Their agricultural value is associated with fruit set, seed output, and crop stability, but their ecological value is associated with the preservation of flowering plant diversity [8,9]. Among bees, the western honeybee, *Apis mellifera*, is especially important for managed crop pollination because colonies can be transported and scaled for agricultural production. Although wild bees and other insects also provide important pollination services [9,10], this review focuses primarily on the western honeybee, *Apis mellifera*, because most available experimental and field evidence on mycotoxin exposure and its effects have been generated from this species. Pollination has a significant economic impact. Animal-mediated pollination significantly increases the market value of numerous fruit, nut, oilseed, and vegetable crops, according to spatial assessments of worldwide crop production [11]. Additionally, studies at the crop level demonstrate that pollinator visitation can enhance production quantity and quality, underscoring the significance of pollinator health beyond crop set [12]. A further reason for the agricultural relevance of honeybees is their capacity for learning and floral cue use. Honeybee foragers can associate floral odors with food rewards, and this sensory learning can influence later flower choice. Experimental work on odor conditioning has shown that in-hive exposure to crop-related odors can bias foraging toward target crops, including pear, apple, almond, sunflower, and kiwifruit systems [13,14,15,16]. Honeybee colony weakening has been associated with several interacting stressors, including habitat loss, agricultural intensification, agrochemical exposure, parasites, pathogens, climate change, and reduced forage diversity [8,17]. A multi-stressor paradigm is typically used to interpret honeybee colony losses. Poor colony performance is frequently linked to pesticides, viruses, *Varroa destructor*, *Nosema* spp., poor nutrition, queen issues, and decreased forage diversity [17,18]. Because pollen provides proteins, lipids, sterols, vitamins, and minerals required for brood rearing, nurse bee physiology, and colony expansion, nutritional stress is particularly important. Poor pollen feeding can weaken colonies, change gut microbiota, impact immunological gene expression, and make them more vulnerable to *N. ceranae* infection, according to field and lab research [19,20]. These findings indicate that pollen quality is a central factor in honeybee nutrition, immunity, and colony resilience.

Most honeybee health research has focused on parasites, pathogens, pesticides, land-use change, and floral resource limitation [17,18]. Many colony health issues are explained by this approach, but it does not fully account for all biologically significant exposure pathways. In addition to direct contact with pesticides or pathogens, honeybees are also exposed through stored colony food. Foragers collect pollen from flowers, transport it in their corbiculae, and deposit it into comb cells, where it is stored and transformed into bee bread. After that, bee bread is mostly eaten by nurse bees and helps the hypopharyngeal glands to produce nourishment for the brood [21]. Contaminants may also enter through this dietary pathway. Pesticide residues, fungal propagules, and fungal secondary metabolites can all be found in pollen gathered by bees [22,23]. Stored pollen is important because it can prolong dietary exposure over time. Pollen contains contaminants that are not specific to the forager who gathered it [24,25]. Through feeding interactions, it may get into the colony’s shared food chain and reach nurses, larvae, and other adult bees [19,21]. As a result, contaminated pollen should be viewed not only as a food source but also as a colony-level exposure matrix. Despite this dietary exposure route, mycotoxins have received much less attention than pesticides in honeybee toxicology. Mycotoxins are typically investigated as pollutants of food and feed, with a focus on exposure to humans and animals [22]. Although direct honeybee evidence is still limited, the pathway is biologically relevant. Saprophytic fungi, including taxa that can produce harmful secondary metabolites, can be found in honeybee nests, which are resource-rich settings [26,27]. Therefore, fungal metabolites should be considered as part of the honeybee dietary exposome.

Mycotoxins are secondary metabolites produced by fungi, including *Aspergillus*, *Penicillium*, and *Fusarium* species [28]. Aflatoxins, ochratoxin A, zearalenone, deoxynivalenol, and T-2 toxin are among the mycotoxins found in bee pollen [29]. Because it is a very poisonous metabolite generated by aflatoxigenic *Aspergillus* species, such as *A. flavus* and related species, aflatoxin B1 is particularly concerning [22,30]. Fungal ecology and honeybee nutrition and toxicology are linked by the presence of aflatoxin-producing fungus in pollen and bee bread [22,23]. Evidence for mycotoxin contamination in bee pollen has increased in recent years. Fungal contamination and measurable aflatoxin B1 were found in Serbian bee pollen samples; all examined samples in that investigation had positive results [31]. Aflatoxin B1 had the greatest incidence of the toxins examined in a subsequent international assessment of commercial bee pollen from 28 countries, which found at least one tested mycotoxin in every sample [23]. Commercial pollen data cannot be directly interpreted as in-hive exposure data for every colony. These results, however, demonstrate that pollen collected by bees might serve as a matrix for the presence of mycotoxins [23,31]. Concern is also supported by direct toxicological findings. Although workers may withstand certain amounts in a lab setting, adult honeybees exposed to aflatoxin B1 and ochratoxin A exhibit quantifiable toxicity [26]. Aflatoxin-producing *A. flavus* metabolites have been linked in more recent research to shorter adult lifespans and smaller hypopharyngeal gland acini in *A. mellifera* workers. Because hypopharyngeal glands are essential for nurse bee function and brood food production, this endpoint is significant [32]. These studies support the need to consider aflatoxins and related fungal metabolites in honeybee health research, although field-realistic colony-level thresholds remain unclear. This concern is also supported by evidence from hive-associated fungi. Bee bread may contain fungal species with possible relevance to colony food ecology, according to an *A. flavus* strain isolated from honeybee bee bread that exhibits characteristics compatible with adaptation to the hive environment. However, because some hive-associated microorganisms may be neutral or context-dependent, the interaction between fungi and bees is not always detrimental [27]. This distinction is crucial. Therefore, the key risk question is not simply whether fungi are present in bee food, but whether fungal contamination results in biologically meaningful mycotoxin exposure.

### 1.1. Scope and Objectives of the Review

This review evaluates mycotoxins as an underappreciated dietary stressor in honeybee health, with emphasis on aflatoxin B1, contaminated pollen, bee bread, and colony-level risk. Four related questions are the main focus of the review. The first is the extent of mycotoxin contamination in pollen and food sources associated with hives. The second is the physiological impacts of aflatoxin or other mycotoxin exposure on honeybees. The third is how the usage of stored pollen, nurse bee function, and brood feeding can scale nutritional exposure from individual workers to colony processes. The fourth is how fungal toxins, together with pesticides, pathogens, and nutritional stress, can be more effectively included in honeybee risk assessment. The main contention is that mycotoxins should not solely be considered a food safety concern for bee products. Additionally, they serve as a conduit of nutritional–toxicological exposure for honeybees. Because pollen is a source of nutrients and a carrier of contaminants, this pathway is conceivable [22,23]. Because pollen nutrition influences immunity, gut microbiota, pathogen susceptibility, and colony strength, it is pertinent [19,20]. Additionally, it can be tested because experimental research has already demonstrated the harmful effects of exposure to aflatoxin on the development of hypopharyngeal glands and the survival of adult workers [26,32]. Therefore, integrating fungal toxins into honeybee health research may improve understanding of colony-level risk, especially under field conditions where poor nutrition, pathogens, pesticides, and microbial contaminants often occur together. The novelty of this review is its honeybee-centered integration of mycotoxin occurrence, pollen and bee bread exposure pathways, nurse bee physiology, brood feeding, and colony-level risk. Previous studies have mainly treated mycotoxins in bee pollen as a food-safety issue or have reported toxicity under laboratory conditions. In contrast, this review connects these separated lines of evidence into a colony-level framework, highlighting how contaminated pollen and bee bread may link fungal metabolites with nurse bee function, larval nutrition, immune responses, gut microbiota, and multi-stressor colony vulnerability. This review is intentionally focused on honeybees (*Apis mellifera*) to avoid overgeneralization across pollinator species and to provide a mechanistic understanding of dietary mycotoxin exposure at colony level. The main output of this review is a conceptual framework describing how mycotoxin-contaminated pollen and bee bread affect honeybee colony health through nurse bee physiology, brood feeding, larval development, gut microbiota, and multi-stressor interactions. Although this review is focused on honeybees (*Apis mellifera*), other bee taxa such as bumble bees, solitary bees, and stingless bees were initially considered but excluded from detailed discussion to maintain a clear and mechanistic focus on honeybee colony-level dietary exposure to mycotoxins.

### 1.2. Literature Search Strategy

This review was conducted as a structured narrative review with scoping-review elements to summarize current evidence on mycotoxin exposure in honeybees and to identify major knowledge gaps. The review was not designed as a meta-analysis because the available studies differ strongly in sample type, toxin type, exposure dose, experimental design, analytical method, and biological endpoint. However, the literature search and selection process were organized transparently to improve reproducibility.

The literature was searched in the Web of Science, Scopus, PubMed, ScienceDirect, SpringerLink, Google Scholar, and MDPI databases. The search covered studies published up to May 2026. The main search terms were used alone and in combination: “honeybee mycotoxin”, “*Apis mellifera* aflatoxin”, “aflatoxin B1 honeybee”, “ochratoxin A honeybee”, “bee pollen mycotoxins”, “bee bread contamination”, “fungal contamination bee pollen”, “mycotoxin-producing fungi pollen”, “deoxynivalenol bee pollen”, “zearalenone bee pollen”, “T-2 toxin bee pollen”, “pollen storage fungi”, “honeybee gut microbiota mycotoxin”, “hypopharyngeal gland honeybee toxin”, and “honeybee colony stressors”.

Articles were included when they met at least one of the following criteria: (i) reported mycotoxin occurrence in bee-collected pollen, bee bread, commercial bee pollen, or hive-related food matrices; (ii) identified mycotoxin-producing fungi associated with pollen, bee bread, or honeybee colonies; (iii) examined biological effects of aflatoxin B1, ochratoxin A, deoxynivalenol, zearalenone, T-2 toxin, or related fungal metabolites on honeybees; (iv) discussed honeybee food pathways, nurse bee physiology, larval nutrition, hypopharyngeal gland development, immunity, gut microbiota, or colony-level stress responses; or (v) provided relevant information on analytical methods for detecting mycotoxins in pollen or bee products. Articles were excluded when they focused only on human food safety without relevance to honeybee exposure; focused only on pesticides or heavy metals without mycotoxins; focused only on general fungal taxonomy without toxin relevance, non-bee insects, or unrelated pollinator decline topics; were duplicated records; were conference abstracts without sufficient data; or were not available in English.

The initial search identified 312 records. After removing duplicates, 247 records were screened by title and abstract. Of these, 118 records were excluded because they were outside the focal topic. A total of 129 full-text articles were assessed, and 31 were excluded because they lacked direct relevance to honeybee mycotoxin exposure, biological effects, or colony-level risk. Finally, 98 articles were included in the review. During revision, 18 additional relevant references were incorporated to address reviewers’ comments and strengthen discussion of exposure pathways, biological effects, analytical methods, and colony-level risk. Thus, the revised manuscript cites a total of 116 references. The selected literature was grouped into five thematic categories: (1) occurrence of mycotoxins and toxigenic fungi in pollen and bee bread; (2) honeybee exposure pathways through pollen, bee bread, and brood food; (3) toxicological and physiological effects on adult bees, nurse bees, larvae, immunity, and gut microbiota; (4) interactions with other stressors, including pesticides, pathogens, poor nutrition, and climate-related fungal contamination; and (5) analytical methods and research gaps for colony-level risk assessment.

## 2. Environmental Origins of Mycotoxins in Honeybee Landscapes

### 2.1. Major Mycotoxin-Producing Fungi

Fungi that invade plants, pollen, stored bee products, and agricultural substrates are the source of mycotoxins found in honeybee pollen and bee bread food supplies. Carbohydrates, proteins, lipids, minerals, and bioactive substances are all found in bee pollen, which is rich in nutrients for honeybee colonies. Additionally, when moisture, temperature, and storage conditions allow fungal development, this composition makes it appropriate for microbial growth [22,33]. *Aspergillus*, *Fusarium*, and *Penicillium* are the primary fungal genera associated with pollen contamination. These genera contain species that can produce fumonisins, zearalenone, trichothecenes, ochratoxin A, and aflatoxins [22,30]. In terms of aflatoxin danger, *Aspergillus* is particularly significant. *Aspergillus flavus* is one of the most researched producers of aflatoxin B1, and the *Aspergillus* section *Flavi* has a number of aflatoxigenic taxa [30,34]. Numerous entrance sites into agricultural and pollinator landscapes are created by the prevalence of *Aspergillus* species in soil, plant debris, seeds, and stored plant materials [30,35]. Fungal dissemination from soil and plant residues to aerial plant parts can occur through spore dispersal and surface colonization, particularly under high humidity and temperature conditions that favor *Aspergillus* growth and aflatoxin biosynthesis [36,37]. Aflatoxin B1 has been found in pollen samples collected by bees, and investigations on bee pollen confirm that *Aspergillus* can grow in pollen matrices [31,35]. Once introduced into the hive environment, pollen can act as a semi-stable matrix where microbial activity and storage conditions may influence fungal persistence and secondary metabolite stability, thereby extending the exposure window for colony members [22]. Despite the environmental ubiquity of aflatoxigenic fungi, acute mortality in honeybees remains uncommon because exposure is typically episodic, low-dose, and diluted across diverse pollen sources, while physiological detoxification systems and gut microbial communities reduce toxic bioavailability [38,39]. As a result, mycotoxin exposure in honeybees is more strongly associated with chronic and sublethal effects rather than immediate lethality. Because honeybee foragers gather pollen from areas where aflatoxigenic fungus may invade crops and floral resources, this connection is crucial. Cereal crops and herbaceous plants are closely related with the primary plant-associated fungus species *Fusarium.* Deoxynivalenol, zearalenone, fumonisins, and other poisons are produced by these species [22,40]. Because pollen can transport poisons and fungal propagules from infected flowers or agricultural plants into the hive, *Fusarium* pollution is relevant to bees [22,33]. *Fusarium* fungi and *Fusarium*-associated mycotoxins, such as zearalenone and deoxynivalenol, have been found in fresh and preserved bee pollen samples under storage settings that encouraged fungal persistence [33]. Because they may colonize food products that have been preserved and create secondary metabolites like ochratoxin A, *Penicillium* species are also important [22,40]. *Penicillium* has a stronger correlation with stored pollen quality in honeybee colony systems than it does with flower field infection. Because bee pollen might get contaminated prior to collection, during hive storage, or after harvest during processing and storage for commercial use, this distinction is important [22,41]. Therefore, post-collection storage conditions and fungal exposure at the landscape level can be represented by the same matrix.

### 2.2. Major Mycotoxins Relevant to Honeybees

Mycotoxins that are frequently found in pollen or that are known to be produced by fungi connected to plant and stored food matrices are the most pertinent to pollinator food supplies. Because they are well-characterized fungal metabolites with recognized toxicological importance in animals, aflatoxin B1, ochratoxin A, deoxynivalenol, zearalenone, and fumonisins are the primary classes of concern [22,40]. Their significance to honeybees is contingent upon their presence in pollen or bee bread, exposure concentration, period of intake, and sensitivity of the exposed bee life stage [23,26].

Since aflatoxin B1 has been found in bee pollen and evaluated directly in honeybees, it is the most obviously relevant to the current review [26,31]. Although honeybee tolerance varies with dose and experimental settings, aflatoxin B1 and ochratoxin A can be harmful to adult honeybees when exposed in a lab setting [26]. Although commercial goods may not necessarily reflect fresh in-hive exposure, commercial pollen surveys also confirm the significance of aflatoxin B1 as a contaminant of bee pollen [23]. These results support the need to pay attention to aflatoxin B1 as a possible dietary toxin for honeybees as well as a food-safety hazard. Although more indirect, the significance of *Fusarium* toxins is nevertheless significant. Bee pollen samples have been found to include deoxynivalenol, zearalenone, and T-2 toxin; storage conditions can affect the presence of these compounds [23,41]. Because *Fusarium* contamination can occur in pollen matrices, fumonisins—well-known *Fusarium* toxins in agricultural commodities—are included in discussions of mycotoxins connected to pollen [22,40]. Since many *Fusarium* toxins still have low direct honeybee poisoning thresholds, their danger should be viewed as an evidentiary gap rather than a proven cause of colony decline.

### 2.3. Climate Change and Fungal Expansion

Because temperature, water activity, humidity, drought stress, and carbon dioxide can change fungal physiology and host vulnerability, climate has a significant role in fungal growth and mycotoxin generation [42,43]. Because honeybees feed on plants whose flowers and pollen develop under the same climatic circumstances that affect fungal colonization, these characteristics are pertinent to pollinator habitats. Therefore, the relationship between climate and bee exposure is indirect but biologically plausible: crops and wild plants are impacted by climate, plants have an impact on the quality and contamination of pollen, and bees gather that pollen into the hive [22,44]. Aflatoxigenic fungi are highly influenced by temperature. Aflatoxin B1 production, fungal growth, and the expression of aflatoxin-related genes can all be altered by interacting climate conditions, according to experiments conducted with *A. flavus* [42]. Battilani et al. (2016) reported that climate warming may increase aflatoxin B1 contamination in maize grain (kernels), indicating that climatic conditions can influence fungal toxin production in major crop systems that may indirectly affect bee-collected floral resources [45]. These results are not unique to honeybees, but they are important for pollinators because flowers, dust, crop residues, and pollen exposed to toxic fungus can all be found in agricultural settings with increased aflatoxin pressure. Another significant factor is drought, as stress on plants can make them more susceptible to toxin buildup and fungal invasion. *Aspergillus* and aflatoxin contamination in crops is particularly affected by drought and high temperatures [43,45]. Additionally, drought can decrease pollen availability and floral diversity, which could make any available pollen source more nutritious for honeybee colonies [17].

Although there are currently few field studies explicitly connecting drought-driven mycotoxin contamination to honeybee colony outcomes, this creates a potential overlap between low nutrition and contaminated pollen exposure. By altering plant damage, postponing harvest, moisture content, and storage quality, extreme weather can also impact the risk of contamination. While storage time and ambient temperature might affect fungal contamination and mycotoxin occurrence in bee pollen, warm and humid conditions can encourage fungal development in stored pollen [41]. The significance of post-collection settings as a component of exposure risk is further supported by commercial bee pollen data, which show that storage temperature is linked to mycotoxin content [23]. These pathways link floral contamination to pollen collected by honeybee foragers. Pollen-based food might have a different contamination profile depending on whether it is stored within or outside the hive. One of the main issues with climate change is geographic redistribution. Changes in the areas where particular fungi become more significant are predicted by climate-change evaluations of mycotoxigenic fungi [43,44]. As warmer weather becomes more conducive to *A. flavus* and aflatoxin B1 buildup, it is anticipated that aflatoxin risk may rise in several temperate crop systems [45]. This is important for managed honeybees since colonies are relocated throughout agricultural areas to facilitate pollination. Therefore, when crops and weather change, mobile colonies may come across shifting fungus and toxin landscapes.

### 2.4. Agricultural Intensification as a Driver

Crop composition, landscape simplification, floral resource concentration, and storage techniques are some of the ways that agricultural intensification might affect mycotoxin exposure [46,47,48]. During critical stages of colony growth, honeybee colonies in simplified agricultural settings frequently rely on mass-flowering crops or few pollen sources [17]. This is significant because, in a diversified landscape, if a single plant species produces a large proportion of the pollen collected by honeybee colonies, contamination in that dominant pollen source can have a stronger effect on overall colony dietary exposure than contamination in less frequently visited plant species. Therefore, the amount of pollen that the colony consumes from infected plants influences the risk in addition to the presence of mycotoxins. Fungal ecology is also impacted by monocultures and landscapes dominated by crops. Crop residues can sustain fungal survival throughout the year, and many mycotoxigenic fungi are linked to important agricultural commodities including grains and oilseed crops [40,43]. Although pollinators do not eat grain, they may come into contact with polluted floral resources or fungal propagules when foraging in these same environments. Since this link is indirect, it should not be used as evidence of colony damage. However, it does pinpoint a plausible environmental source for fungal metabolites seen in pollen gathered from agricultural systems. Honeybee colonies may experience brief times of intensive pollen collecting due to mass-flowering crops. When pollen is plentiful and varied, this pattern can improve colony nutrition; however, when the available pollen source contains residues or microbial pollutants, it can also concentrate exposure [17,22]. Several mycotoxins, such as aflatoxin B1, ochratoxin A, deoxynivalenol, zearalenone, and T-2 toxin, can be found in bee pollen, according to commercial pollen surveys [23]. The necessity to assess chemical and fungal pollutants within the same pollen matrix is reflected in the use of both pesticides and mycotoxins in multi-contaminant methodologies for bee pollen [49]. Because pollen is not only ingested during collecting, storage-associated contamination is particularly significant for honeybees. Hive-stored pollen is incorporated into the colony’s food chain when foragers deposit pollen into comb cells. The microbiological quality of pollen can be affected by temperature, moisture content, fungal load, and length of storage [22,41]. Research on stored bee pollen reveals that lengthy or warm storage conditions can alter fungal composition and mycotoxin detection [41,50]. These results imply that mycotoxins are not limited to fields and flowers. Contamination can occur during flowering, hive storage, or post-harvest handling. Mycotoxin exposure in pollinator landscapes follows a continuum from soil to stored pollen through fungal contamination. Despite the presence of continuous environmental sources of fungal propagules and mycotoxins across soil, plants, and stored materials, honeybee mortality associated with these compounds remains relatively uncommon. This is primarily because exposure levels in natural foraging conditions are typically low, intermittent, and highly variable, and contaminated pollen is frequently diluted within multi-floral diets at the colony level [39]. In addition, honeybee detoxification systems mediated by cytochrome P450 enzymes, together with gut microbiota-assisted metabolic processing, reduce the effective bioavailability of ingested toxins [38,51]. As a result, mycotoxin exposure in honeybees is more strongly associated with chronic and sublethal physiological effects rather than acute mortality or colony collapse [39,51]. Soil, plants, crops, flowers, and stored substrates are the sources of fungi. Toxins or fungal propagules may then enter the honeybee’s food chain through pollen. Each stage of this route can be altered by climate change and agricultural intensification, although the evidence now available is strongest for the prevalence of contamination and weakest for the effects on field-scale colonies [22,23].

### 2.5. Analytical Detection of Mycotoxins

Determining the precise levels of mycotoxins in pollen and bee bread is crucial for evaluating pollinators’ nutritional intake. Because of its repeatability, separation effectiveness, and compatibility with complicated matrices, high-performance liquid chromatography (HPLC) is frequently used for the detection of aflatoxin and other mycotoxins. Aflatoxin B1 in bee pollen and associated products has been successfully measured using HPLC with fluorescence detection or post-column derivatization. Although HPLC techniques offer accurate quantification and low detection limits, they frequently include thorough sample preparation, including extraction, purification, and cleanup using solid-phase extraction or immunoaffinity columns, which can add variability and lengthen processing times [52]. High sensitivity and selectivity for several mycotoxins at once are provided by liquid chromatography combined with tandem mass spectrometry (LC-MS/MS). This method can handle co-eluting chemicals and complex matrices typical of pollen and bee bread, and it enables the simultaneous detection of aflatoxins, ochratoxin A, fumonisins, and trichothecenes in a single run [53]. Additionally, structural validation of analytes is provided by LC-MS/MS, improving quantification reliability. However, in order to avoid matrix effects interfering with accuracy, LC-MS/MS needs costly equipment, knowledgeable operators, and strict calibration. It is regarded as the gold standard for multi-residue analysis in apicultural products in spite of these difficulties [23]. Because of its ease of use, speed, and capacity to handle large sample sets, the enzyme-linked immunosorbent assay (ELISA) is widely used for screening. Commercially available ELISA assays for ochratoxin A and aflatoxin B1 have been verified for bee pollen matrices [54]. ELISA provides semi-quantitative results with moderate sensitivity and is suitable for preliminary surveillance. Limitations include cross-reactivity with structurally related mycotoxins, matrix interference, and lower precision compared with chromatographic techniques, necessitating confirmatory testing with HPLC or LC-MS/MS. ELISA remains useful for high-throughput monitoring when resources or equipment access are limited. The precision and repeatability of all analytical techniques are greatly impacted by sample preparation. The molecular complexity of bee pollen and bee bread includes proteins, lipids, carbohydrates, and secondary metabolites that may make identification difficult. To enable accurate quantification, extraction solvents, cleanup techniques, and sample homogenization must be carefully tailored for each toxin class [41]. Method validation and inter-laboratory standardization are essential because comparative studies have demonstrated that the choice of analytical method affects observed concentrations [31]. When combined, these methods offer complementary ways to identify and measure mycotoxins, aiding risk analysis and exposure assessment in pollinator health research. The analytical methods commonly used for detecting mycotoxins in pollen and bee bread, including HPLC-FLD, LC-MS/MS, ELISA, and GC-MS/MS, are summarized in Table 1.

## 3. Exposure Pathways: How Honeybees Encounter Mycotoxins

### 3.1. Floral Contamination

Through food sources that start at the flower and eventually make their way into the nest, honeybees come into contact with fungal metabolites. Flowers are not sterile organisms. Their nectar, pollen, floral surfaces, and reproductive tissues can harbor bacterial and fungal communities that are influenced by the environment, animal visits, and plant characteristics [56,57]. Since pollen is the primary source of protein for *Apis mellifera* and is gathered directly from anthers during foraging, fungal colonization of floral resources is relevant to honeybees [22]. Foragers may move toxic fungus or their metabolites from pollen grains into corbicular pollen burdens and subsequently into colony food stores [23,31]. Floral microorganisms can be spread throughout flowers by visiting animals or introduced prior to pollinator visits. Fungal communities can be supported by nectar and floral surfaces, and microbial assembly in floral nectar can be influenced by visitor identification [58,59]. These results do not demonstrate that mycotoxins are produced by all floral fungi. They demonstrate how flowers might serve as microbial interfaces between pollinators and plants. When fungi like *Aspergillus*, *Fusarium*, or *Penicillium* are present in pollen-associated matrices, this interface is important for mycotoxin exposure [22,33]. Aflatoxin B1, ochratoxin A, deoxynivalenol, zearalenone, and T-2 toxin can be found in pollen, according to bee pollen surveys, although the source could be field contamination, hive storage, or post-harvest management [23,31].

### 3.2. Foraging-Mediated Exposure

Floral contamination is transformed into food exposure through foraging. In corbicular loads, honeybee foragers gather pollen from several plant types and deliver it to the hive. Fungal propagules and contaminated pollen particles from flowers may enter the colony as a result of this behavior. Because foragers do not instantly swallow all obtained pollen, the exposure route is biologically significant. A large portion of the pollen is deposited into comb cells, where it is accessible to developing brood and nurse bees [21].

When it comes to pollen-based mycotoxin exposure, honeybees are the most researched pollinator group. Adult honeybees exhibit dose-dependent sensitivity to aflatoxin B1 and ochratoxin A under laboratory feeding conditions, and aflatoxin B1 has been found in pollen collected by bees [26,31]. Aflatoxin B1 is commonly found in examined samples, and commercial pollen surveys provide additional evidence that bee pollen can include many mycotoxins [23]. An exposure pathway is supported by this research. Field-realistic danger levels for colonies have not yet been established.

Although there is still a lack of direct mycotoxin evidence for these taxa, Bumble bees can affect the microbial makeup of flowers by feeding on blooms that have microbial communities [57,58]. Bacterial and fungal symbionts derived from plants, nests, and the environment are found in the pollen-nectar supplies that solitary bees store for their larvae [60,61]. These findings demonstrate the potential for exposure across bee taxa. They do not prove that managed honeybees and wild bees have the same toxicological effects or mycotoxin dosages.

### 3.3. Bee Bread as a Chronic Exposure Reservoir

Because it connects short-term foraging events with longer-term food consumption, bee bread is essential for colony exposure. Pollen is combined with nectar, honey, and bee secretions in wax cells that are deposited by honeybee foragers [21]. The term “fermented bee bread” is frequently used to describe this preserved pollen. Hive-stored pollen, however, is best characterized as a conserved food matrix rather than a system intended for substantial microbial nutrient conversion, according to thorough microbiological and functional investigations [21,62]. This distinction is crucial for mycotoxins because, while preservation may inhibit microbial growth, it does not always remove toxins that are already present in pollen. When contaminated pollen stays in comb cells and is eventually digested, bee bread can serve as a reservoir for prolonged exposure. One international assessment found that commercial bee bread had greater total mycotoxin concentrations than other pollen formats; however, processing and storage circumstances outside of the hive may have an impact on commercial products [23]. According to storage studies, fungal contamination and mycotoxin presence in bee pollen can be influenced by temperature, storage time, moisture, and fungal load [33,41]. *Fusarium* fungi and toxins linked to *Fusarium* can be found in both fresh and stored bee pollen, supporting the idea that storage conditions can influence contaminant profiles after collection [33]. Pollen that has been preserved has a complicated microbial ecology. The microbial composition of fresh and stored bee pollen can vary, and preservation may encourage bacteria that can withstand acidic and sugar-rich environments [50,62]. This does not imply that mycotoxins are always present in bee bread. Fungal species, water activity, temperature, oxygen availability, substrate quality, and storage time all affect toxin generation and persistence [22,41]. Bee bread can prolong exposure from a single contaminated foraging episode into a recurring food route for nursing bees, which is crucial for colony risk.

### 3.4. Larval Exposure

Through nurse bee feeding, honeybee larvae are indirectly exposed to elements generated from pollen. Through the secretions of their mandibular and hypopharyngeal glands, nurse bees absorb the nutrients from stored pollen to make brood food [63]. Later worker and drone larvae are fed meals that contain more material obtained from pollen, while young larvae are fed glandular brood food [64]. Because nursing bees can metabolize or buffer chemicals before brood food is released, this feeding system can lessen direct larval interaction with some pollutants [64,65]. Additionally, when pollutants or nutritional changes brought on by contaminants enter the larval diet, they may generate an exposure pathway. Although pesticide behavior cannot be presumed to mirror mycotoxin behavior, evidence from pesticide research is helpful in understanding the route. The quantity of pollen grains in larval feeding rises with larval age, and worker jelly may contain residues from pesticide-contaminated pollen meals [64]. Pesticide residues may be present in royal jelly, and contamination may come from nectar or pollen gathered from flowers that have been treated [65]. These results demonstrate that polluted forage supplies might be linked to larvae diets through colony food transfer. There are currently few comparable transfer studies for aflatoxin B1 and other mycotoxins in brood feeding. Larvae may be indirectly impacted by aflatoxin’s effects on nurse bees. *Aspergillus mellifera* workers who were exposed to metabolites and aflatoxins from *Aspergillus flavus* had shorter lifespans and smaller hypopharyngeal gland acini [32]. The condition of the hypopharyngeal glands is important because they produce brood food that is high in protein [63]. Thus, two questions about larval exposure are supported by current research. The first is whether brood food is contaminated with mycotoxins. The second is whether the quantity or quality of brood food produced by nurse bees exposed to mycotoxins is affected.

### 3.5. Queen Exposure

Worker feeding, colony food dynamics, and royal jelly can all lead to queen exposure. Workers feed the adult queen, and during their growth, queen larvae are fed copious amounts of royal jelly [65,66]. This makes it possible for tainted pollen to indirectly impact queen nutrition through nurse bees. Even in cases where residue transmission is minimal, pesticide studies demonstrate that colony-level exposure to contaminated pollen can modify the composition of royal jelly, including alterations in proteins, metabolites, and sterols. Because it distinguishes between direct contaminant transport and contaminant impacts on nurse bee physiology and glandular production, this pathway is pertinent to mycotoxin study. There is currently little concrete proof that royal jelly exposes queens to aflatoxin B1. Consequently, rather than being a predetermined result, reproductive implications should be considered a research priority. The evidence that is now available warrants caution because worker care, colony nutritional status, and larval diet quality all affect queen growth and reproductive performance [66]. Queen feeding may be indirectly impacted if mycotoxins hinder nursing bee survival or hypopharyngeal gland growth [32]. Mycotoxin levels in royal jelly, queen-destined larval diet, and queen tissues must be specifically measured in order to test this idea.

### 3.6. Colony-Level Exposure Dynamics

**Figure 1:**
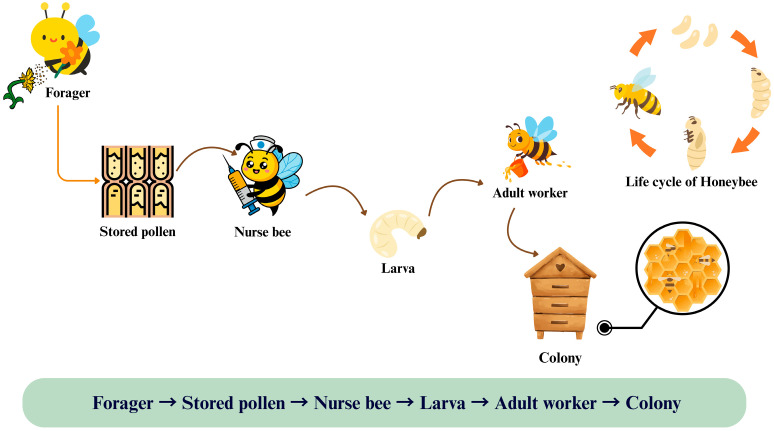
Conceptual pathway of mycotoxin transmission and trophic transfer within honeybee colonies. Contaminated pollen collected by forager bees is stored as pollen or bee bread and subsequently transferred through nurse bees to larvae and adult workers, ultimately influencing colony-level health and resilience. The diagram highlights the progression of exposure across key biological compartments involved in honeybee development, nutrition, and colony maintenance.

Rather than being a single-bee occurrence, mycotoxin exposure in honeybee colonies should be understood as a food-system process. Although a forager may initially come into contact with a contaminated pollen grain, the primary risk pathway emerges once nurse bees store and ingest the pollen. Then, through trophic interactions and brood feeding, nurse bees link stored pollen to larvae, adult workers, and queens [21,64]. This structure can buffer or dilute exposure. It also distributes nutritional stress across colony processes. Figure 1 illustrates the conceptual pathway of mycotoxin transmission within a honeybee colony, beginning with contaminated pollen collection by foragers and progressing through trophic interactions involving nurse bees, larvae, adult workers, and ultimately colony-level outcomes.

The primary biological compartments where measurements are required are identified by this approach. Exposure to the landscape is defined by foragers. Reservoir exposure is defined by stored pollen and bee food. The generation of food for brood and physiological processing is defined by nurse bees. Developmental exposure is defined by larvae. Downstream effects on workforce size and performance are defined by adult workers. The colony establishes the ultimate threshold at which exposure can impact worker replacement, resilience, and brood production. Pollen pollution and laboratory toxicity to adult honeybees have the strongest evidence currently available [23,26]. There is only minimal evidence of mycotoxin and fungal profile alterations brought on by storage [33,41]. Field-realistic colony results are still not well supported. This gap is crucial because pollen contamination alone cannot determine colony-level risk. Toxin concentrations in pollen, nurse bees, bee bread, brood food, larvae, and adult workers under realistic colony conditions must be linked.

### 3.7. Environmental Concentrations vs. Toxicological Thresholds

By comparing observed amounts in pollen and bee bread with levels used in honeybee toxicity tests, it is possible to infer environmental mycotoxin exposure in the pollinator diet. Aflatoxin B1 is found at low μg/kg quantities in a number of areas, according to bee pollen surveys, although the results differ significantly between nations, pollen kinds, storage circumstances, and analytical techniques. Aflatoxin B1 was found in all samples of Serbian bee pollen, with a mean value of 8.61 μg/kg [31]. Mold and aflatoxin contamination were also found in bee-collected pollen in a subsequent Serbian investigation, demonstrating that contamination can happen in pollen that honeybees directly gather [67]. On the other hand, after immunoaffinity cleanup and HPLC-FLD analysis, aflatoxins were not detectable in Chinese commercial bee pollen samples, which had a variety of fungus communities [54]. While aflatoxin B2, aflatoxin G1, aflatoxin G2, fumonisin B1, and citrinin remained below detection limits, Turkish bee products exhibited mean aflatoxin B1 of 0.595 μg/kg and fumonisin B2 of 2.091 μg/kg throughout studied bee products [68]. According to these studies, risk evaluation should use measured regional concentrations rather than assuming constant exposure because pollen contamination is not uniform.

Because bee bread is stored colony nourishment and can extend exposure beyond a single foraging excursion, it needs special management. In a multi-country assessment that included aflatoxin B1, ochratoxin A, zearalenone, deoxynivalenol, and T-2 toxin, commercial bee bread had greater overall mycotoxin contents than fresh and dried bee pollen [23]. This distinction matters because bee bread is mostly eaten by nurse bees and is associated with the manufacture of brood food. However, because harvesting, drying, moisture content, and storage temperature can alter fungal development and toxin buildup, commercial bee bread could not accurately reflect in-hive bee bread. This warning is supported by storage studies, which found that temperature and storage time had an impact on mycotoxin occurrence and fungal contamination in bee pollen [41]. *Fusarium* fungus and *Fusarium*-associated mycotoxins were also found in fresh bee pollen that was briefly stored, demonstrating that contamination might alter after pollen collection [33]. Therefore, before determining colony-level danger, bee bread should be measured directly within colonies.

There are primarily two types of laboratory toxicity data for honeybees: ochratoxin A and aflatoxin B1. Under laboratory conditions, adult *A. mellifera* tolerated dietary aflatoxin B1 in a 1 and 2.5 μg/g diet; however, toxicity increased at greater exposure levels and after cytochrome P450 activity was inhibited [26]. These tested low dosages are equivalent to a 1000 and 2500 μg/kg diet, which are significantly greater than the majority of pollen concentrations that have been measured. The 1 μg/g laboratory dose is approximately 116 times greater than the mean Serbian pollen concentration of 8.61 μg/kg, while the 2.5 μg/g dose is approximately 290 times higher. The 1 μg/g dose is almost 1680 times greater than the mean aflatoxin B1 level of 0.595 μg/kg in Turkish bee products [26,31,68]. These straightforward ratios indicate a wide-seeming gap between short-term laboratory dosages and numerous outdoor pollen quantities. Because most laboratory testing did not adequately account for chronic bee bread exposure, nurse bee sensitivity, mixed mycotoxins, pesticides, nutritional stress, and pathogen pressure, this margin should not be considered a safety threshold.

For sublethal endpoints, the margin of exposure is very ambiguous. *Aspergillus flavus* metabolites that produce aflatoxin decreased the lifespan of honeybees and the size of hypopharyngeal gland acini, indicating a connection between nurse bee physiology and fungal exposure [32]. Honeybee midgut structure, transcriptome responses, and microbial profiles were all impacted by sublethal aflatoxin B1 exposure, suggesting that non-lethal dosages can nevertheless have an impact on physiologically significant tissues [69]. These results suggest that colony-relevant impacts may be underestimated by mortality-based thresholds alone. Additionally, analytical investigations demonstrate that various techniques identify distinct toxin classes and concentration ranges. *Fusarium*-related mycotoxins were found in bee pollen using GC-MS/MS [53]. Aflatoxin and ochratoxin A sensitivities in bee pollen were enhanced by LC-MS/MS techniques [52]. Extensive studies employing ELISA or chromatographic techniques demonstrate that matrix type, toxin class, and sample processing affect detection [23]. The most reasonable conclusion is that current environmental concentrations are frequently below acute laboratory toxicity doses. However, because dose–response data for nurse bees, larvae, queens, and entire colonies have not been matched with chronic exposure through bee bread, the true colony-level margin is still unknown.

## 4. Toxicological Effects of Mycotoxins on Honeybees

### 4.1. Survival and Longevity

Adult workers and a few fungal compounds continue to be the focus of direct toxicological data in honeybees. Under laboratory conditions, adult *A. mellifera* workers can withstand low dietary concentrations of aflatoxin B1 and ochratoxin; however, when the exposure concentration increases or metabolic detoxification is impeded, mortality increases [26]. This trend suggests that aflatoxin B1 is both a biologically active dietary toxicant for honeybees and a contamination of pollen resources [22]. According to the same study, cytochrome P450 involvement was connected to enhanced aflatoxin B1 toxicity following piperonyl butoxide exposure, placing detoxifying capacity at the core of honeybee tolerance to this mycotoxin [26]. Because nurse bees and immature workers frequently eat pollen and bee bread, chronic exposure is more important for colonies than acute exposure. Adult worker longevity was shortened by experimental exposure to metabolites from aflatoxigenic *A. flavus*, and this was linked to smaller hypopharyngeal gland acini in *A. mellifera* [32]. Because nurse bee glands create royal jelly for queens and protein-rich brood food for larvae, this endpoint is relevant to the colony [63]. Therefore, a survival-risk route focused on adult workers, particularly nurses, is supported by the available honeybee data; nevertheless, field-based fatal limits for contaminated pollen or bee bread have not yet been established [26,32]. Although there is significant taxonomic heterogeneity, evidence from insects other than honeybees supports the biological plausibility of mycotoxin poisoning. Mycotoxin effects vary by insect order, species, developmental stage, toxin type, and diet composition, according to a systematic analysis of insect studies [70]. Compared to lepidopteran and dipteran insects, coleopteran insects frequently exhibit greater resistance to aflatoxin B1, and later larval stages are frequently less vulnerable than early stages [70]. These distinctions caution against drawing conclusions about honeybees directly from non-bee insects. Additionally, they demonstrate that before colony-level risk can be measured, honeybee-specific life stage assays are required.

### 4.2. Developmental Effects

There is a significant information gap about developmental toxicity in honeybees. Unlike adult workers, honeybee larvae do not eat raw pollen. Nurse bees provide glandular brood food to young larvae, while older worker larvae receive food that contains more material obtained from pollen. This feeding method may lessen direct larval exposure to certain toxins, but it can also transfer nutritional stress through nurse bee physiology and brood food composition [63,64]. Larval exposure to aflatoxin B1 is still not fully understood since mycotoxin transfer into brood diet has not been described with the same level of detail as pesticide transfer. Mycotoxins can impact growth and survival during juvenile stages, according to research on insects other than honeybees; however, reactions differ greatly between species and toxins. Because honeybee larvae are rapidly growing insects with a high nutritional need, this evidence is significant. It cannot, however, be regarded as concrete evidence of *A. mellifera* brood toxicity [70]. Indirect development is the biggest concern for honeybees. Brood feeding may be impacted before direct larval toxicity is even detectable if exposure to aflatoxin reduces nurse bee lifetime or hypopharyngeal gland development [32,63]. Because pollen provides the amino acids, lipids, sterols, vitamins, and minerals required for larval growth and worker replacement, pollen quality also affects brood development. In honeybees, nutritional stress can weaken colonies, change gut microbiota, and make them more vulnerable to *N. ceranae* infection [19,20]. Contaminated pollen may therefore act through two interconnected pathways: direct toxin exposure and decreased nutritional function of the pollen-based diet. This is not a proven field mechanism of brood failure, but it is supported by current research as a priority hypothesis [22,23].

### 4.3. Behavioral Effects

In contrast to the behavioral toxicology of pesticides, the behavioral toxicology of mycotoxins in pollinators is less developed. Foraging, navigation, learning, and social communication have not received as much attention in the literature on insect mycotoxins as survival, growth, toxin buildup, and biotransformation [70]. Because colony performance depends on coordinated foraging, dance communication, orientation flights, food exchange, and age-related task allocation, this creates a significant gap for honeybees [63,64]. There is currently little concrete proof that aflatoxin B1 or other mycotoxins affect honeybee foraging, learning, navigation, or waggle-dance communication. Indirect and physiology-based behavioral concerns are the most tenable. Reduced hypopharyngeal gland development can impact nurse function, and decreased adult longevity can change the age structure of workers [32,63]. Field research is required before mycotoxins can be ascribed a specific role in communication disruption or foraging impairment, even if these effects may have an impact on colony labor balance. It is incorrect to assume that there is no danger in the absence of robust behavioral evidence. In other toxicological circumstances, honeybee behavioral endpoints are extremely sensitive markers of sublethal stress, particularly when exposure comes from food [64,66]. Thus, learning assays, orientation measurements, eating behavior, trophallaxis, and foraging return rate should all be included in mycotoxin research. Because survival alone might overlook colony-relevant sublethal effects, these endpoints are essential.

### 4.4. Reproductive Effects

The effects of mycotoxins on honeybee reproduction are still mostly unknown. Larval diet, the quality of royal jelly, worker care, successful mating, and continuous adult feeding by workers all affect queen health [66,71]. Proteins, amino acids, lipids, carbohydrates, vitamins, and minerals that support queen development and larvae feeding are found in royal jelly, which is made by nurse bees [63,71]. Queen-related nutrition may therefore be indirectly impacted by any food toxicant that modifies nurse bee gland function; however, this pathway has not been formally evaluated for aflatoxin B1 in honeybees [32]. Colony food contamination can reach queen-relevant feeding pathways, as evidenced by research on pesticide exposure that demonstrates how contaminated colony meals can change royal jelly production and nutritional composition [66]. Because mycotoxin metabolism and pesticide chemistry are different, this evidence cannot be directly applied to mycotoxins. However, it recognizes royal jelly as a crucial matrix for upcoming monitoring of ochratoxin A and aflatoxin [22,66]. Another understudied endpoint is drone fertility. Male honeybees exhibit a unique sensitivity to physiological stress, and drones are crucial for queen mating success and colony genetic continuity. Drone survival may be impacted by oxidative stress, and workers and drones express stress-response proteins differently [72,73]. There is currently insufficient direct evidence of mycotoxin impacts on drone sperm viability, semen volume, or mating performance. Therefore, following realistic contaminated pollen or bee bread meals, future research should assess drone exposure, sperm quality, testis growth, and oxidative stress.

### 4.5. Physiological Biomarkers

The most reliable method for linking mycotoxin exposure to colony-relevant outcomes is through physiological indicators. Aflatoxin B1 toxicity rises when P450 activity is reduced, and cytochrome P450 monooxygenases are essential for honeybee detoxification of several xenobiotics [26,74]. As a result, P450 activity and detoxifying gene expression are significant indicators of honeybee exposure to aflatoxin. Under coupled stress, the same route may also influence whether nutritional exposure stays sublethal or advances toward mortality. Because mycotoxins can cause cellular damage in animal systems and oxidative balance is crucial for honeybee life and male stress tolerance, oxidative stress is particularly significant [40,73]. Therefore, lipid peroxidation, antioxidant enzyme activity, protein carbonylation, and glutathione-related reactions should be included in honeybee-specific aflatoxin research. These biomarkers are frequently employed in the physiology of insect stress and are associated with immunological status, tissue condition, and survival [73]. One crucial element that is missing is tissue pathology. One direct tissue-level endpoint for mycotoxin-related stress in workers is decreased hypopharyngeal gland acini size in adult honeybees exposed to aflatoxin-producing *A. flavus* metabolites [32]. Additionally, the midgut should be given priority since it is essential for digestion, detoxification, and host–microbe interactions and is the first tissue exposed to contaminated pollen or bee bread after ingestion [20,70]. Residue analysis, histopathology, detoxification indicators, oxidative stress markers, microbiome profiling, and colony performance metrics should all be included in future toxicological investigations. Because chronic food exposure, rather than isolated acute toxicity, shapes honeybee risk, an integrated strategy is required [19,26,32]. The major physiological effects associated with mycotoxin exposure in honey bees, including impacts on survival, hypopharyngeal glands, midgut structure, detoxification capacity, gut microbiota, and immune function, are summarized in Table 2.

## 5. Mycotoxins, Immunity, and Disease Susceptibility

### 5.1. Pollinator Immune Systems

Both communal and individual immune systems are essential for honeybees. Epithelial barriers, antimicrobial peptides, melanization, cellular responses, RNA interference, and stress responses associated with detoxification are all examples of individual immunity [78,79]. By using sanitary practices, grooming, antimicrobial nest materials, and colony-level evacuation of sick brood, social immunity lowers the spread of pathogens [80]. Although these defenses work well, they are expensive in terms of energy. Nutrition, age, pathogen pressure, and exposure to environmental toxins all have a significant impact on their performance [20,81]. Because pollen provides amino acids, lipids, sterols, vitamins, and minerals required for immunological signaling, gland growth, and tissue repair, pollen nutrition and immune competence are tightly related [25,81]. In honeybees, nutritional stress can change the gut microbiome, lower immune-related reactions, and enhance *N. ceranae* infection [20]. Additionally, under field settings, nutritional stress at the colony level decreases adult and brood bee numbers and increases susceptibility to disease [19]. Because of this, contaminated pollen is crucial for studying immunity. It can transport fungal toxins and serve as a source of nutrients [22,23].

### 5.2. Immunosuppressive Potential of Aflatoxins

A biologically active fungal metabolite, aflatoxin B1, has been shown to have immunotoxin effects in animal systems [76,82]. Dose, length of exposure, host species, tissue, and physiological state all affect its immunological effects [76]. Dietary aflatoxin B1 can damage barrier tissues, change inflammatory signaling, inhibit immune-related gene expression, and lower infection resistance in vertebrates [82,83]. These effects demonstrate that aflatoxin B1 can interfere with immunological modulation in animals following oral exposure, although they are not particular to honeybees [76,84]. Insect evidence is still limited yet pertinent. Aflatoxin B1 exposure changed the expression of genes linked to immunity and metabolism in black soldier fly larvae, suggesting a quantifiable transcriptome response to this toxin in an insect model [77]. Under laboratory feeding settings, aflatoxin B1 is toxic to adult honeybees, and its toxicity is increased when cytochrome P450 activity is inhibited [26]. This suggests that honeybee tolerance to aflatoxin B1 is influenced by detoxifying capacity [26,74]. There is still little concrete proof that aflatoxin B1 inhibits honeybee immune systems. Targeted research on antimicrobial peptide expression, melanization, phenoloxidase activity, RNA interference, gut barrier integrity, and pathogen replication following realistic pollen-based exposure is justified by the available data [26,77].

### 5.3. Interactions with Pathogens

The host’s diet and intestinal health have a significant impact on *N. ceranae*, a significant microsporidian parasite of adult honeybees [20,85]. In a lab setting, nutritional stress can enhance *N. ceranae* infection and alter the gut microbiome and immunological responses of honeybees [20]. Reduced colony strength and changed disease patterns are also observed in field colonies under nutritional stress [19]. Testing mycotoxin–*Nosema* interactions is strongly justified by these findings. While *Nosema* develops in the adult gut, where nutrition, bacteria, and immunology interact, mycotoxins can enter through pollen and bee bread [20,22]. Another significant axis of disease is represented by viruses. Numerous RNA viruses, such as the Israeli acute paralysis virus, sacbrood virus, black queen cell virus, and deformed wing virus, are found in honeybees [79,86]. Nutrition, co-exposure to chemical stressors, *Varroa destructor* pressure, host immunological status, and viral load all influence the severity of viral illness [86,87]. In honeybees, sublethal pesticide exposure can worsen immunological modulation and boost viral replication, demonstrating how environmental or dietary toxicants might alter pathogen outcomes [87]. There are currently no comparable trials using aflatoxin B1. This is a crucial gap because young workers who maintain colony immunological stability and brood care may eat pollen infected with aflatoxin [21,26]. Attention should also be paid to bacterial illnesses. Honeybee larvae that consume spores are killed by American foulbrood, which is caused by *Paenibacillus larvae* [88]. *Melissococcus plutonius* is the cause of European foulbrood, which has an impact on brood health through nutritional disturbance and larval infection [89]. As of right now, there is no solid proof that exposure to aflatoxin B1 increases the frequency of foulbrood in honeybee colonies. However, colony hygiene practices, nurse bee care, and larval diet all affect brood illnesses [88,89]. Therefore, mycotoxin effects on nurse bee longevity or hypopharyngeal gland development may be pertinent to the ecology of brood diseases, although this pathway has not yet been well investigated in colonies [32,63].

### 5.4. The Mycotoxin–Pathogen Synergy Hypothesis

According to the mycotoxin–pathogen synergy concept, tainted pollen may increase the risk of disease when it coexists with pathogen pressure and nutritional stress. Three streams of evidence support this theory. First, mycotoxins such as aflatoxin B1 can be found in pollen and bee bread [23,31]. Second, under regulated exposure, aflatoxin B1 is harmful to adult honeybees, and other mycotoxins can impact immunological modulation and disease susceptibility in animals [26,76]. Third, inadequate pollen nutrition can change immunology, gut microbiome, and *Nosema* infection, making honeybees more susceptible to illness [20,81]. The suggested method is straightforward and verifiable. Mycotoxins may increase the cost of tissue healing and detoxification. The resources available for glandular activity and immunological function may be diminished by nutritional stress. The physiological and energetic demands on adult workers and brood may be increased by pathogens. The combined impact of these stressors may be greater than that of each stressor acting alone [81,90]. However, field evidence for colony-level effects remains limited. It indicates that they fit into a multi-stressor paradigm that is biologically plausible but has not been sufficiently taken into account in research on honeybee diseases. Therefore, mycotoxin exposure through pollen or bee bread, decreased immune regulation or detoxification capacity in adult workers, altered gut microbiota and epithelial condition, increased pathogen replication or persistence, decreased nurse bee performance, impaired brood support, and decreased colony resilience provide a useful framework for future research. Direct measurement is necessary at every stage. Toxin residues in bee bread, nurse bee tissues, and brood food; immune gene expression; gut microbiota structure; *Nosema* intensity; virus load; brood survival; and colony expansion should be the top goals. It will continue to be challenging to distinguish mycotoxin contamination from poor nutrition, pesticide exposure, and pathogen pressure in the absence of these linked metrics.

### 5.5. Detoxification and Defense Mechanisms Against Mycotoxins in Honeybees

Honeybees possess multiple physiological, microbial, and behavioral defense mechanisms that contribute to tolerance against dietary mycotoxins. At the biochemical level, detoxification is primarily mediated by enzyme systems such as cytochrome P450 monooxygenases, glutathione S-transferases, and carboxylesterases, which are involved in the metabolism and neutralization of xenobiotic compounds [51,91]. These pathways reduce the bioavailability and toxicity of ingested fungal metabolites, including aflatoxins and related secondary compounds.

In addition to enzymatic detoxification, the honeybee gut microbiota plays an essential role in maintaining host resilience by contributing to metabolic processing, immune modulation, and suppression of harmful microbial activity. Symbiotic bacteria such as *Gilliamella apicola* and *Snodgrassella alvi* are involved in carbohydrate metabolism and chemical detoxification processes that indirectly influence host tolerance to dietary stressors [92]. Furthermore, behavioral mechanisms such as selective foraging, nectar and pollen mixing, and dilution of contaminated resources within diverse floral diets reduce the effective exposure concentration of mycotoxins at the colony level.

These combined defense strategies provide a mechanistic explanation for the relatively low acute mortality of honeybees observed under field conditions, despite experimental evidence of sublethal effects of mycotoxins on immunity, gut integrity, and glandular function. Therefore, honeybee responses to mycotoxins should be interpreted primarily in terms of chronic and sublethal colony-level effects rather than acute lethality.

## 6. Gut Microbiota Disruption as a Mechanistic Link

### 6.1. Importance of Bee Gut Symbionts

The hindgut of an adult honeybee is home to a specialized and very basic bacterial community that is dominated by host-adapted lineages [93]. Adult worker bees and social transmission among colonies are frequently linked to core bacterial taxa such as *Snodgrassella alvi*, *Gilliamella apicola*, *Bifidobacterium*, *Lactobacillus Firm-4*, and *Lactobacillus Firm-5* [93,94]. The honeybee’s diet is chemically complex, rich in pollen, and heavily reliant on plant sources, making this microbial system crucial. Gut symbionts aid in the metabolism of pollen-derived substrates, the fermentation of carbohydrates, the synthesis of organic acid, and the breakdown of secondary chemicals in plants [95,96]. Through bacterial metabolism and host endocrine signaling, which connects microbial activity with adult worker growth and nutritional physiology, the honeybee gut microbiome also encourages host weight increase [97]. Since the gut is the initial internal contact where pollen nutrients, fungal metabolites, and microbial symbionts interact after ingestion, these functions are pertinent to contaminated pollen [22,94]. Disease resistance is also influenced by the gut microbiome. Under experimental conditions, antibiotic alteration of the honeybee gut microbiota increases susceptibility to opportunistic bacterial infection and decreases survivorship [98]. A direct function for gut symbionts in colonization resistance is supported by the ability of native bee gut bacteria to shield workers from *Serratia marcescens* invasion [99]. The gut microbiome is at the core of diet–immunity–pathogen interactions in honeybees because nutritional stress can change immunological responses, gut microbiota, and *N. ceranae* infection [20].

### 6.2. Mycotoxin-Induced Dysbiosis

There is currently little concrete proof of mycotoxin-induced dysbiosis in honeybees. This gap is significant because honeybee gut symbionts are known to react to chemical and nutritional stresses, and aflatoxin B1 and other mycotoxins have been found in bee pollen [22,23]. Under laboratory feeding conditions, adult honeybees exposed to aflatoxin B1 exhibit toxicity; however, microbiome-level responses were not resolved in the same study [26]. Adult worker lifespan and hypopharyngeal gland acini size were decreased by more recent exposure to aflatoxin-producing *A. flavus* compounds; nevertheless, microbiota consequences are still not well understood [32]. The biological plausibility of mycotoxin-driven intestinal dysbiosis is supported by data from various animal systems. Because intestinal bacteria can change, bind, or metabolize mycotoxins and because toxins can change the composition of microbes and the function of the gut barrier, mycotoxins and gut microbiota interact in both directions [75]. In monogastric animals, aflatoxin B1 has an impact on immunological responses, oxidative balance, intestinal microbiota, epithelial shape, and nutritional digestion [100]. Mycotoxin exposure is linked to barrier degradation and dysbiosis in a food-consuming animal model by causing intestinal inflammation, disrupting tight junctions, and altering gut microbial structure in laying hens [101]. Evidence from insects supports caution as well; however, it should not be applied too broadly to honeybees. Mycotoxin effects differ by insect species, life stage, nutrition, and toxin class, according to a comprehensive assessment of insect studies [70]. Consumption of mycotoxin-producing fungi impacted fitness and changed the composition of gut bacteria in a study on the soil arthropod *Folsomia candida*, suggesting that fungal toxins can affect gut-associated bacteria in invertebrates [55]. Following exposure to aflatoxin B1, ochratoxin A, deoxynivalenol, and mixed mycotoxins at pollen-relevant quantities, these results confirm the necessity of honeybee-specific microbiome assays [23,26].

### 6.3. Consequences for Nutrition and Immunity

Honeybee health can be impacted by gut microbiota disturbance through feeding before obvious toxicity manifests. In order to help the host digest nutrients, honeybee gut bacteria metabolize substrates derived from pollen and create fermentation products [95,96]. Young adult workers’ microbiota-dependent weight growth demonstrates that gut symbionts affect host nutritional physiology in ways other than basic digesting [97]. Therefore, exposure to fungal toxins and disruption of microbial processes that facilitate pollen usage may be two interrelated stressors associated with a contaminated pollen diet [22,75]. The effects on the immune system are very significant. Honeybee gut dysbiosis can lower survival following microbial challenge and increase susceptibility to opportunistic bacterial diseases [98,99]. Because glyphosate treatment changes the gut microbiome of honeybees and enhances their vulnerability to *S. marcescens* infection under experimental settings, it offers a helpful analogy [102]. Additionally, exposure to glyphosate can change immunological responses, such as melanization-related defenses and the expression of antimicrobial peptides, and decrease good gut bacteria [103]. These results demonstrate that gut microbiome change can link dietary chemical exposure to immunological dysfunction in honeybees; however, they do not demonstrate a similar response for aflatoxin B1 [102,103]. This mechanism is further complicated by nutritional stress. Honeybee gut microbiome, immunity, and the severity of *N. ceranae* infection are all affected by poor pollen nutrition [20]. Under field-relevant conditions, nutritional stress at the colony level also weakens the colony and alters disease-related outcomes [19]. Therefore, mycotoxin-contaminated pollen should be assessed not only for its toxin content but also for its impact on the gut microbiota and the pollen’s nutritional value [22,23].

### 6.4. The Gut–Immune–Colony Axis

A mechanistic underpinning for associating contaminated pollen with colony-level risk is provided by the gut–immune–colony axis. According to this theory, contaminated pollen enters the colony through foragers, turns into stored pollen or bee bread, and is mostly eaten by young adult workers and nursing bees [21]. These workers rely on immunological competence for resistance to gut pathogens and systemic infection, as well as on gut microbial processes for pollen processing [94,99]. The gut microbial composition, intestinal barrier condition, detoxification activity, and immune gene expression should be the first observable consequences if mycotoxins disrupt gut microbiota or intestinal function [75,100]. For nursing bees, this axis is especially important. Aflatoxin-producing *A. flavus* metabolites can decrease the size of hypopharyngeal gland acini in adult workers, and nurse bees use hypopharyngeal gland activity to transform pollen-derived nutrients into brood food [32,63]. A gut-level disturbance may therefore scale to colony function through decreased food absorption, altered immunological state, shorter worker longevity, and lower brood-feeding capacity [20,32]. Because there are still few direct studies linking aflatoxin exposure, intestinal dysbiosis, brood food quality, and colony growth, these connections are still partially inferential for mycotoxins [23,26].

Experimental use is the most effective application of this framework. Mycotoxin residues in pollen, bee bread, gastrointestinal contents, nurse bees, and brood food in the same colonies should be measured in future research. Microbiome profiling, intestinal histology, immunological indicators, pathogen load, nurse bee gland development, brood survival, and colony expansion should all be paired with residue data in these investigations. This system would distinguish between exposure that is biologically significant and simple pollution. Additionally, it would make clear if mycotoxins primarily affect the honeybee colony’s food system as direct toxicants, microbiome disruptors, nutritional modifiers, or interacting stressors.

## 7. Multi-Stressor Interactions: The Missing Dimension

### 7.1. Mycotoxins × Pesticides

**Figure 2:**
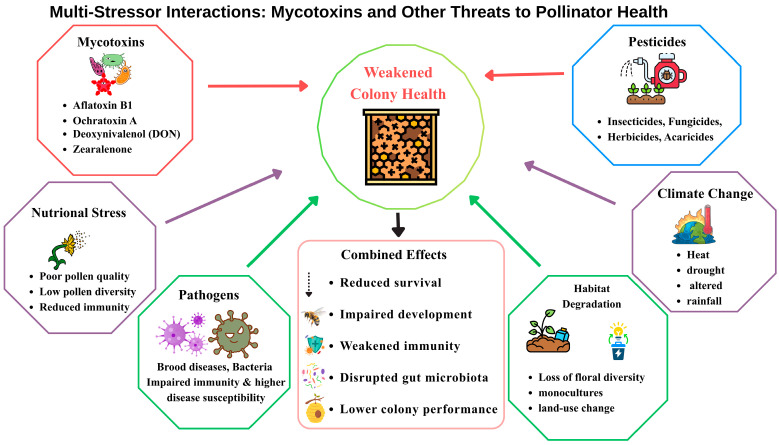
Illustration of multi-stressor interactions affecting honeybee colony health. Mycotoxins in pollen and bee bread interact with pesticides, nutritional stress, climate change, pathogens, and habitat degradation, collectively weakening colony survival, development, immunity, and gut microbiota. The diagram emphasizes the combined impact of these stressors and highlights the need for integrated colony-level risk assessment.

Honeybee colonies are commonly exposed to multiple co-occurring stressors in agricultural environments [17]. Multiple pesticide residues, such as insecticides, fungicides, herbicides, and acaricides used inside hives, can be found in pollen and wax [104]. Mycotoxins such as aflatoxin B1, ochratoxin A, deoxynivalenol, zearalenone, and T-2 toxin can also be found in bee pollen [22,23]. According to these results, mycotoxins and pesticides belong to the same dietary exposure matrix. Young workers and nursing bees frequently eat stored pollen and bee bread, which makes this overlap significant [21]. As shown in Figure 2, which indicates mycotoxins in pollen, an underappreciated threat to pollinator health is present. On the other hand, a mechanistic basis for interaction is conceivable. Many xenobiotics are detoxified by adult honeybees using glutathione S-transferases, cytochrome P450 monooxygenases, and other metabolic processes [74]. Inhibiting cytochrome P450 activity increases the toxicity of aflatoxin B1, suggesting that detoxifying capacity affects honeybee tolerance to this fungal toxin [26]. Additionally, a number of pesticides rely on detoxification routes, and certain pesticide combinations can be more hazardous to bees than individual substances [104,105]. This suggests a shared detoxification pathway for both pesticides and mycotoxins in honeybees. Although fungicides are frequently thought to be less acutely harmful to bees than insecticides, they can change the toxicity of pesticides through metabolic interactions; therefore, they warrant particular consideration [105]. Fungicide use may also alter fungal communities on plants, pollen, and stored bee food, which makes this pertinent to mycotoxins. The direction of this interaction depends on fungal competition and pesticide-induced changes in pollen microbiota. Fungicides may change microbial competition in pollen matrices, select for resistant fungi, or inhibit some fungal development [22]. Therefore, rather than treating pesticide residues and mycotoxins as distinct exposure groups, risk assessment should quantify them jointly.

### 7.2. Mycotoxins × Nutritional Stress

One of the main factors influencing honeybee health is pollen quality. Immune system performance, physiology, and disease resistance are influenced by pollen diversity and nutritional composition [25,81]. Under field conditions, nutritional stress weakens colonies and modifies disease-related outcomes [19]. Additionally, it impacts immunological indicators, gut microbiome, and *N. ceranae* infection in adult workers [20]. Poor nutrition is a possible amplifier of dietary toxicants due to these effects. There are two possible interactions between nutritional stress and mycotoxin-contaminated pollen. First, despite offering insufficient or unbalanced nutrients, contaminated pollen may expose workers to fungal toxins [22,23]. Second, undernourished bees may be less able to withstand toxic exposure since detoxification and tissue regeneration need metabolic resources [26,81]. Because nurse bees need pollen to sustain hypopharyngeal gland function and brood food production, this connection is particularly important for them [63]. Adult worker lifespan and hypopharyngeal gland acini size can be decreased by exposure to *A. flavus* metabolites and aflatoxins [32]. This discovery connects a tissue that is crucial for brood care with a polluted diet. Whether polluted pollen becomes a modest exposure or a substantial colony stressor also depends on the nutritional situation. While colonies in simplified landscapes might mainly rely on fewer pollen sources, colonies foraging in diverse landscapes might dilute tainted pollen with other floral resources [17]. This does not imply that all pollen with low nutritional value is tainted. Because they both function through the same dietary pathway, nutritional restriction and contamination should be examined simultaneously.

### 7.3. Mycotoxins × Climate Change

By changing fungal growth, plant stress, and toxin production, climate change can raise the risk of mycotoxins. The growth of toxic fungi and the generation of aflatoxins and other mycotoxins are influenced by temperature, water activity, drought, humidity, and carbon dioxide [42,43]. In regions of Europe where current conditions have historically been less conducive for aflatoxin accumulation, model-based research suggests that warming may raise the likelihood of aflatoxin B1 contamination in maize [45]. These studies indicate landscape-level factors that may affect pollen contamination, but they are not honeybee experiments. Heat exposure, changed blooming phenology, drought-induced feed restriction, and decreased floral continuity are other ways that climate stress impacts pollinators [17,106]. When hot and dry conditions raise the risk of fungal toxins while simultaneously decreasing the amount of feed available, these pressures may converge with mycotoxin exposure. Colonies may experience lesser pollen diversity, polluted pollen, and increased physiological stress under such circumstances [43,81]. Because honeybee colonies rely on constant worker replacement and brood feeding during growth phases, this convergence is crucial. Post-collection exposure may also be impacted by extreme weather. Temperature, length of storage, moisture, and fungal contamination all affect stored bee pollen. In pollen matrices, warm storage may promote mycotoxin incidence and fungal persistence [41]. This results in two climate-linked pathways for managed colonies: contaminated pollen gathered from stressed landscapes and modified fungal dynamics in stored pollen or additional pollen products.

### 7.4. Mycotoxins × Pathogens

Host nutrition, immunology, microbiome, and co-exposure to toxicants all influence the impact of pathogens, which are important causes of honeybee colony loss [17,86]. Workers or developing bees may suffer significant physiological expenses due to *N. ceranae*, viruses, and brood infections [85,88,89]. In mature honeybees, nutritional stress can change immunological responses and promote *N. ceranae* infection [20]. Pesticide exposure can also interact with wax and raise mortality throughout the honeybee life cycle [90]. Through food, gut physiology, and immune modulation, mycotoxins may fit within this pathogen paradigm. Under laboratory feeding conditions, aflatoxin B1 is harmful to adult honeybees, and mycotoxins can impact animal systems’ immune systems [26,76]. Additionally, the honeybee stomach plays a crucial role in pathogen contact, microbial balance, and nutrition metabolism [94]. Contaminated pollen may modify a host’s susceptibility to opportunistic bacteria or *Nosema* if it modifies the gut or immune system. Because there are still few direct honeybee investigations that combine aflatoxin B1 with viruses, Nosema, or brood diseases, this theory continues to be a priority. Because immunological disturbance can affect viral replication, virus interactions are particularly important. Exposure to clothianidin can hinder the regulation of the antiviral immune system and encourage the multiplication of the deformed wing virus in honeybees [87]. This pesticide example demonstrates how a dietary or environmental toxin can alter the course of a pathogen. Viral load, immune gene expression, gut barrier status, and survival should all be assessed simultaneously when testing mycotoxins.

### 7.5. Mycotoxins × Habitat Degradation

By decreasing floral diversity and making colonies rely on fewer pollen sources, habitat degradation can raise the relevance of mycotoxins. Reduced fodder availability is associated with poor nutrition and decreased colony performance, and loss of flower-rich environments is a significant factor in pollinator reduction [8,17]. In agricultural settings, land-use patterns can affect the availability of resources and the survival of honeybee colonies [107]. Contaminated pollen from a dominating crop or a restricted floral source may make up a greater portion of the colony’s diet in deteriorated settings. The likelihood that honeybees will face several stressors in one area is likewise increased by agricultural expansion. Bees in crop-dominated systems may be exposed to fungal pollutants in plant-derived food matrices, diseases from densely managed colonies, pesticides, and nutritionally limited pollen sources [22,104]. The necessity to assess contaminated pollen as part of the larger agricultural exposome is supported by commercial bee pollen surveys, which reveal that pollen can include numerous mycotoxins [23]. Habitat loss increases reliance on contaminated or low-diversity pollen sources due to reduced floral diversity and forage availability. It might decrease dietary buffering and increase reliance on tainted resources. The existence of a single novel toxin class is not the missing dimension in pollinator stress studies. Pollen contamination, chemical exposure, malnutrition, climate stress, infections, and damaged forage areas all interact to cause this. Thus, mycotoxins should be investigated as dietary toxicants that interact. This method can determine the circumstances in which aflatoxin-contaminated pollen represents a hazard to honeybee resistance and distinguish between simple contamination and biologically significant colony harm [23,108,109].

## 8. Why Current Pollinator Risk Assessments Fail to Capture Mycotoxins

### 8.1. Regulatory Focus on Pesticides

Pesticide exposure, acute toxicity, and residue-based hazard estimation in honeybees have been the primary focus of pollinator risk assessment [110,111]. Because pesticides are regularly found in pollen, wax, nectar, and hive matrices and are purposefully administered in agricultural settings, this approach makes scientific sense [104,112]. However, toxicants that enter colonies through the same food channel and are either naturally generated or related to storage may be overlooked by a pesticide-centered paradigm. Because fungi create mycotoxins instead of applying them as agrochemicals, they belong in this neglected category [22]. Pollen can carry both fungal toxins and chemical residues, according to available data. Wax, pollen, and bee samples from North American apiaries showed a variety of pesticide residues [104]. Aflatoxin B1, ochratoxin A, deoxynivalenol, zearalenone, and T-2 toxin were among the mycotoxins found in commercial bee pollen from several nations [23]. These results suggest that a pollen-based diet exposes honeybee colonies to a variety of contamination profiles. However, mycotoxin surveillance and pesticide risk assessment have often emerged as distinct fields of study [22,110]. The evaluation of contaminated pollen as a combined nutritional and toxicological exposure matrix is limited by this separation.

### 8.2. Absence of Mycotoxins in Honeybee Testing Protocols

Pesticide toxicity testing, exposure through treated crops, residue transfer, and species extrapolation from *Apis mellifera* to non-*Apis* bees are highlighted in peer-reviewed research on pollinator risk assessment [110,111]. Despite proof that aflatoxin B1 and ochratoxin A are hazardous to adult honeybees when fed in a lab, these methods hardly ever include mycotoxins as standard test materials [26]. Because mycotoxins are dietary toxicants, this absence is crucial. Instead of spray drift or direct contact exposure, they can infiltrate colonies through pollen and stored food [22,23]. There are many technical limitations due to the absence of mycotoxin-specific methods. While mycotoxin risk may rely on nurse bees’ long-term consumption, persistence in bee bread, and potential impacts on brood food production, standard bee toxicology frequently prioritizes adult acute death [21,32]. The requirement for endpoints beyond mortality is supported by the fact that adult honeybee exposure to aflatoxin-producing *A. flavus* metabolites decreased longevity and hypopharyngeal gland acini size [32]. Additionally, fungal toxin combinations receive little consideration in current testing frameworks. This is a drawback, since pollen samples may simultaneously contain several mycotoxins [23].

### 8.3. Limitations of Existing Exposure Models

Fungal toxin dynamics in pollen and bee bread cannot be quantified by current exposure models, although they do capture significant aspects of bee ecology. Colony demography, feeding habits, landscape resources, *Varroa* infection, viruses, and colony failure mechanisms are all connected by BEEHAVE. BEEHAVE is a mechanistic, individual-based simulation model developed to study honeybee colony dynamics by integrating key biological and environmental processes, including foraging behavior, colony demography, resource availability, pathogen pressure, and colony losses under different ecological conditions. It is widely used to evaluate how multiple stressors such as nutrition, landscape composition, and parasite infection interact to influence colony performance over time [113]. Understanding how colony processes react to interrelated stressors is made easier with the help of this kind of model.

It does not, however, specifically depict mycotoxin synthesis, fungal colonization of pollen, toxin persistence in stored pollen, or nutritional transfer from nurse bees to larvae [22,113]. Additionally, the transferability of residue-based pesticide exposure models to mycotoxins is restricted. Application rate, crop attractiveness, residue content, and bee foraging behavior are frequently used to quantify pesticide exposure [110,111]. Other factors that affect mycotoxin exposure include fungal species, plant infection, temperature, water activity, pollen moisture, storage duration, and handling after collection [22,41]. Assessments of pollinator exposure seldom take these factors into account. Because of this, while existing models can identify contaminated pollen, they do not explain when contamination becomes biologically significant for nurses, larvae, queens, or colony expansion. Another restriction is species coverage. Although solitary bees and bumble bees have different nesting biology, larval provisioning, food range, and exposure routes, pesticide risk assessment has traditionally heavily depended on honeybees [111,114]. This issue relates to mycotoxins as well. While honeybee larvae are fed by nurse bees and may indirectly acquire pollutants through brood food, solitary bee larvae eat sealed pollen supplies [21,111]. Therefore, hazards for wild bees without colony-level dilution and worker-mediated buffering may be underestimated by a honeybee-only exposure model.

### 8.4. Need for Integrated Risk Assessment

**Figure 3:**
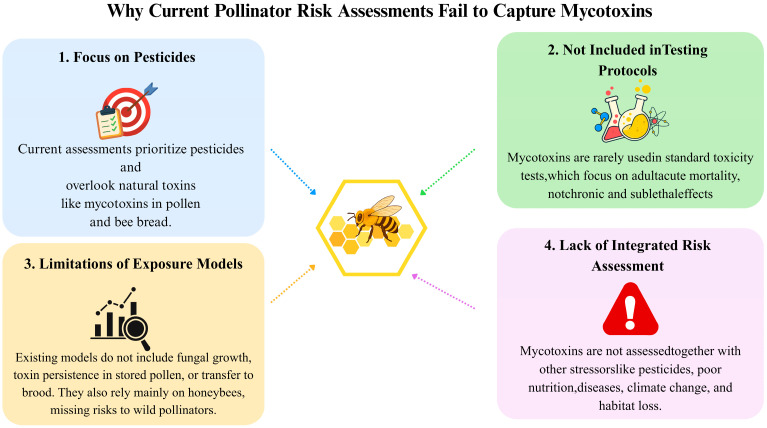
Simplified diagram illustrating why current pollinator risk assessments fail to capture mycotoxins. Key gaps include a pesticide-centered focus, absence of mycotoxins in standard testing protocols, limitations of existing exposure models, and lack of integrated, multi-stressor risk assessment. The figure emphasizes that dietary fungal toxins, interacting with other stressors, remain a hidden threat to honeybee and pollinator colony health.

Using an integrated dietary-exposure paradigm, mycotoxins should be included in pollinator risk assessment. Pollen and bee bread should be viewed within this paradigm as matrices including nutrients, fungal propagules, pesticide residues, and fungal metabolites [22,23,104]. Additionally, it should link exposure to biological effect levels that are important for colony function. These include pathogen burden, immunological markers, gut microbiome, hypopharyngeal gland development, nurse bee survival, brood survival, and colony population dynamics [19,20,32]. Mycotoxins should not be assumed to act alone in an integrated assessment. Pesticides, infections, nutritional stress, climate stress, and landscape degradation all affect honeybee colonies concurrently [17,110]. Detoxification capability affects aflatoxin B1 toxicity in honeybees, and detoxification pathways can also interact with pesticide combinations [26,74]. This is in favor of combined-exposure approaches that evaluate mycotoxins in conjunction with viral illness, *Nosema*, pesticides, and inadequate pollen feeding. These investigations would shed light on whether mycotoxins are significant amplifiers of colony stress or insignificant background pollutants. Mycotoxin residues in pollen and bee bread, toxin persistence during storage, internal exposure in adult workers and brood feeding, sublethal physiological effects, and colony-level outcomes should all be included in a feasible risk-assessment model. The field would go beyond occurrence surveys with this structure. Additionally, it would enable risk assessors to separate contaminated pollen from contaminated pollen that is important to toxicology. Mycotoxins enter colonies through one of the most significant pathways in honeybee biology, yet without this integration, they will remain outside the primary pollinator danger paradigm. For clear understanding of all these, Figure 3 is a simplified conceptual diagram illustrating major limitations of current pollinator risk assessment frameworks, including their pesticide-centered focus, exclusion of mycotoxins from standard testing protocols, inadequate exposure models, and limited consideration of multi-stressor interactions affecting honeybee and pollinator colony health.

## 9. Research Priorities for the Next Decade

### 9.1. Global Surveillance of Pollen and Bee Bread Mycotoxins

Coordinated monitoring of mycotoxins in pollen supplements, bee bread, and pollen collected by bees is the top focus. Aflatoxin B1 and other mycotoxins can be found in bee pollen, according to the available data; however, sampling varies by geography, season, floral source, and hive matrix [22,23,31]. Commercial goods may reflect both in-hive exposure and post-harvest handling and storage; however, commercial pollen surveys offer valuable occurrence data [23]. Therefore, corbicular pollen, freshly stored pollen, mature bee bread, and supplemental pollen products should be separated during field surveillance [21,41]. Toxins and fungal taxa should be measured in the same samples during surveillance. This is required since toxin detection does not necessarily reveal the initial source of contamination, and the presence of *Aspergillus*, *Fusarium*, or *Penicillium* does not always confirm toxin production [22,30]. Because honeybees eat pollen as a mixed chemical and nutritional matrix, multi-analyte techniques that identify pesticides and mycotoxins in bee pollen are particularly crucial [49,104]. Toxin concentration, moisture, storage conditions, pollen botanical origin, colony state, and landscape setting should all be included in standard residue reporting [23,41].

### 9.2. Species-Specific Toxicity Thresholds

Determining species-specific toxicity thresholds is the second priority. According to honeybee data, adult workers may be impacted by aflatoxin B1 and ochratoxin A when fed in a lab; nevertheless, the existing thresholds are still insufficient for risk assessment at the field level [26]. Adult worker lifetime and hypopharyngeal gland acini size can be decreased by exposure to *A. flavus* metabolites and aflatoxins, indicating the necessity for sublethal endpoints in addition to mortality [32]. Thus, adult survival, nurse bee gland growth, eating behavior, immunological indicators, detoxification biomarkers, and brood-relevant endpoints should all be considered thresholds [32,63]. Additionally, thresholds need to go beyond *Apis mellifera*. Colony size, larval feeding, diet breadth, nesting biology, and microbial reliance are all different amongst solitary bees, stingless bees, and bumble bees [111,115,116]. Extrapolation from honeybees to other pollinators is limited since insect responses to mycotoxins differ significantly by species, life stage, food, and toxin class [70]. Therefore, representative social and solitary bee species should be included in risk assessments, and assays should be created based on their natural eating biology [111].

### 9.3. Chronic Low-Dose Exposure Studies

Research on chronic low-dose exposure is the third priority. Short acute assays are unable to fully capture the biological significance of dietary mycotoxins since honeybee colonies frequently consume pollen and bee bread [21,22]. When occurrence data indicate co-contamination, field-relevant concentrations, actual pollen or bee bread matrices, and mixed mycotoxin profiles should be used in chronic exposure studies [23,26]. Because colony-level risk may result from decreased nurse bee performance, altered gut function, or poor brood care rather than rapid adult death, low-dose designs are crucial [19,32]. Combined exposures should also be included in chronic tests. Honeybees frequently deal with pesticides, nutritional stress, infections, and poor pasture conditions [17,90]. These stressors might interact to raise mortality or disease burden. Testing mycotoxins with pesticide mixes that share or obstruct detoxification pathways is supported by the fact that aflatoxin B1 toxicity in honeybees is impacted by detoxification capacity [26,74]. Therefore, mycotoxins alone and in conjunction with pesticides, inadequate pollen nutrition, *N. ceranae*, and viral infection should be assessed in long-term studies [20,86].

### 9.4. Colony-Level Experimental Studies

Colony-level experimentation is the fourth priority. While individual assays are helpful in identifying hazards, food storage, nurse bee physiology, brood feeding, worker replacement, and colony demography, all influence honeybee risk [21,113]. Mycotoxin investigations should shift from cage assays to nucleus colonies, observation hives, and field colonies with measured exposure in pollen, bee bread, adult workers, brood food, and larvae [32,64]. Brood area, adult bee population, queen status, food reserves, disease load, worker longevity, and foraging activity should all be monitored in colony-level investigations [19,107]. Because tainted pollen does not always indicate colony damage, this step is crucial. A colony may avoid certain resources, metabolize poisons, dilute contaminated pollen, or buffer exposure through communal feeding [21,94]. When contaminated pollen is kept, ingested by nurse bees, and associated with inadequate nutrition or pathogen pressure, the opposite result may also occur [20,32]. To determine which circumstances turn residue detection into quantifiable colony risk, colony-level research is required.

### 9.5. Microbiome-Mediated Effects

The function of gut microbiota is the fifth priority. A unique gut bacterial population found in adult honeybees aids in metabolism, nutrition, and disease resistance [93,94]. The gut is at the core of diet-mediated disease vulnerability because nutritional stress can change immunity, gut microbiota, and *N. ceranae* infection [20]. In animal systems, mycotoxins interact with intestinal microbiota to modify immune responses, gut barrier function, and toxin metabolism [75]. Research on honeybees should examine if gut microbial structure or function is disrupted by aflatoxin B1, ochratoxin A, deoxynivalenol, zearalenone, or combined mycotoxins. Toxin residues should be combined with 16S rRNA sequencing, metagenomics, gut histology, immunological markers, detoxification markers, and pathogen burden in these investigations [98,99]. This approach would make it clear whether mycotoxins primarily cause immunological disruption, intestinal dysbiosis, decreased nutritional utilization, direct toxicity, or a combination of these effects [20,75].

### 9.6. Climate Driven Forecasting Models

Predicting mycotoxin exposure under climatic change is the sixth priority. Fungal growth and mycotoxin production are influenced by temperature, humidity, water activity, drought, and carbon dioxide [42,43]. According to modeling, in areas where the existing conditions are less conducive to aflatoxin accumulation, warming may raise the danger of aflatoxin B1 in crops [45]. Because honeybees feed in the same environments where agricultural and floral fungal contamination can alter, these climate-driven shifts are important for pollinators [22,44]. Climate variables, crop distribution, flowering phenology, pollen availability, fungal ecology, toxin occurrence, and colony foraging behavior should all be incorporated into forecasting models. Fungal toxin generation and toxin dynamics in bee bread are not explicitly represented by current colony models, but they can mimic honeybee demography, foraging, parasites, viruses, and colony failure [113]. Risk projections for particular geographies, seasons, crops, and management strategies would be possible with the addition of mycotoxin modules. Residue data from pollen and bee bread obtained under known climate and floral circumstances should be used to validate these models [23,41].

### 9.7. Inclusion in Honeybee Risk-Assessment Frameworks

**Figure 4:**
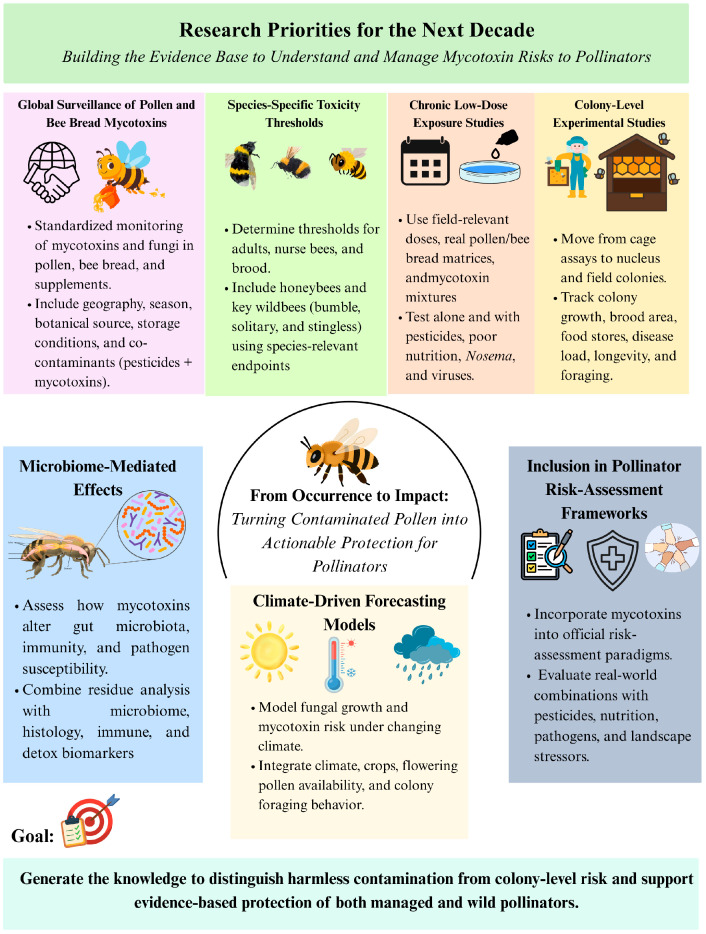
Infographic illustrating key research priorities for addressing mycotoxin risks in pollinators over the next decade. Centralized around a bee and honeycomb graphic, the diagram highlights seven interconnected focus areas: global surveillance of pollen and bee bread, species-specific toxicity thresholds, chronic low-dose exposure studies, colony-level experiments, microbiome-mediated effects, climate-driven forecasting models, and inclusion in integrated pollinator risk assessment frameworks. This visual emphasizes the need for coordinated, multi-scale, and mechanistic research to transform occurrence data into actionable strategies that protect both managed and wild pollinator populations.

The official inclusion of mycotoxins in pollinator risk assessment is the last priority. While fungal toxins are still not routinely tested by pollinators, current frameworks have a major emphasis on pesticide exposure, pesticide toxicity, and species extrapolation [110,111]. Since mycotoxins can be found in bee pollen and adult honeybees exhibit detectable sensitivity to aflatoxin B1 under controlled exposure, this omission is no longer warranted [23,26]. Instead of being considered a distinct food safety concern, mycotoxins should be assessed as dietary contaminants of pollen-based food resources [22]. Occurrence, exposure, toxicokinetic, sublethal physiology, microbiome impacts, pathogen interactions, and colony performance should all be included in an integrated framework. Additionally, since honeybee colonies encounter these pressures collectively in agricultural environments, it should evaluate realistic combinations of mycotoxins, pesticides, nutritional stress, and infections [17,90,104]. It would not be assumed that mycotoxins are the primary cause of colony loss if they were included in the risk assessment. It would establish the evidentiary framework required to distinguish between contaminated pollen that is an innocuous background exposure and that which becomes a stressor at the colony level. Figure 4 is an infographic illustrating major research priorities for addressing mycotoxin risks in pollinators over the next decade, emphasizing integrated surveillance, mechanistic toxicology, colony-level studies, microbiome interactions, climate-driven forecasting, and risk assessment frameworks for both managed and wild pollinators.

## 10. Conclusions

Mycotoxins are an emerging but under-recognized dietary stressor in honeybee health. This review highlights that pollen and bee bread act as key exposure pathways for fungal metabolites such as aflatoxin B1, ochratoxin A, deoxynivalenol, and zearalenone. Once ingested, these compounds can affect nurse bee physiology, brood food production, larval development, immune function, and gut microbiota, thereby influencing overall colony performance. The evidence indicates that mycotoxin effects alone are unlikely to cause colony collapse but may significantly amplify honeybee vulnerability when combined with other stressors, including poor nutrition, pathogens, pesticides, and environmental pressures. However, a major limitation in current research is the disconnect between measured mycotoxin residues in hive matrices and their biological impacts at the colony level.

This review proposes a honeybee-centered framework linking dietary exposure through pollen and bee bread to colony-level physiological and functional outcomes. This framework integrates key biological endpoints, including hypopharyngeal gland activity, larval nutrition, immune responses, microbiome stability, and colony productivity, to improve risk interpretation. Future research should move beyond simple detection studies and focus on mechanistic, multi-stressor, colony-level experiments that quantify exposure and link it directly to biological outcomes. Such studies will improve understanding of how dietary fungal toxins contribute to honeybee decline under real field conditions. Overall, mycotoxins should be considered an integral component of the honeybee dietary exposome, alongside pesticides, pathogens, and nutritional stress, with direct relevance to colony health and resilience.

## Figures and Tables

**Table 1:** Analytical methods for mycotoxin detection in pollen and bee bread.

Method	Mycotoxins Detected	Advantages	Limitations	References
HPLC-FLD	Aflatoxin B1	Accurate quantification; low detection limits; reproducible	Extensive sample preparation; time-consuming	[22,31,55]
LC-MS/MS	Aflatoxins, OTA, DON, ZEA, T-2, fumonisins	Multi-toxin detection; high sensitivity; structural confirmation	Expensive equipment; skilled operator required; matrix effects	[23,52,53]
ELISA	Aflatoxin B1, OTA	Rapid; high-throughput; low cost; portable	Semi-quantitative; cross-reactivity; confirmatory testing needed	[23,54]
GC-MS/MS	DON, ZEA, T-2 toxin	Excellent separation for *Fusarium* toxins	Derivatization required; less suitable for aflatoxins	[23,53]

Abbreviations: HPLC-FLD, high-performance liquid chromatography with fluorescence detection; LC-MS/MS, liquid chromatography–tandem mass spectrometry; ELISA, enzyme-linked immunosorbent assay; GC-MS/MS, gas chromatography–tandem mass spectrometry; OTA, ochratoxin A; DON, deoxynivalenol; ZEA, zearalenone.

**Table 2:** Physiological effects of mycotoxin exposure on honeybees (*Apis mellifera*).

Endpoint	Observed Effect	Toxin	Life Stage	References
Survival	Dose-dependent mortality; reduced adult lifespan	AFB1, OTA	Adult worker	[26,27]
Hypopharyngeal gland	Reduced acini size; impaired brood food production	AFB1 or *A. flavus* metabolites	Nurse bee	[32,63]
Midgut	Altered histomorphology; transcriptome changes	AFB1	Adult worker	[69]
Detoxification capacity	Increased toxicity when P450 is inhibited	AFB1	Adult worker	[26,74]
Gut microbiota	Altered microbial composition (dysbiosis)	AFB1	Adult worker	[69,75]
Immune function	Changed antimicrobial peptide expression	AFB1	Adult worker	[76,77]

Abbreviations: AFB1, aflatoxin B1; OTA, ochratoxin A; *A. flavus*, *Aspergillus flavus*; P450, cytochrome P450 monooxygenase.

## Data Availability

Not applicable.

## Abbreviations

AFB1 (Aflatoxin B1); DON (Deoxynivalenol); OTA (Ochratoxin A); ZEA (Zearalenone); P450 (Cytochrome P450 monooxygenase); HPG (Hypopharyngeal gland); *N. ceranae* (*Nosema ceranae*); *P. larvae* (*Paenibacillus larvae*); *S. alvi* (*Snodgrassella alvi*); *G. apicola* (*Gilliamella apicola*); *S. marcescens* (*Serratia marcescens*); *A. flavus* (*Aspergillus flavus*); *V. destructor* (*Varroa destructor*); RNAi (RNA interference); AMP (Antimicrobial peptide); PO (Phenoloxidase); GST (Glutathione S-transferase); DWV (Deformed wing virus); IAPV (Israeli acute paralysis virus); BQCV (Black queen cell virus); EFB (European foulbrood); AFB (American foulbrood); LC-MS/MS (Liquid chromatography–tandem mass spectrometry); ELISA (Enzyme-linked immunosorbent assay); GC-MS/MS (Gas chromatography–tandem mass spectrometry).
